# The conceptual/procedural distinction belongs to strategies, not tasks: A comment on Gabriel et al. (2013)

**DOI:** 10.3389/fpsyg.2013.00820

**Published:** 2013-11-06

**Authors:** Thomas J. Faulkenberry

**Affiliations:** Department of Psychology and Counseling, Tarleton State UniversityStephenville, TX, USA

**Keywords:** fractions, conceptual knowledge, procedural knowledge, strategies, mathematics education

In their recent article, Gabriel et al. (2013) propose that the difficulty experienced by Belgian children in learning fractions stems from the fundamental dichotomy of procedural and conceptual knowledge of fractions. Indeed, the authors are not alone in this conclusion, as the procedural/conceptual knowledge divide has been a focus in several recent studies in numerical cognition (Hallett et al., 2010; Hecht and Vagi, 2012). Like their predecessors, Gabriel et al. adopt the definitions introduced by Rittle-Johnson and Alibali (1999), where conceptual knowledge refers to the understanding of the principles that govern a knowledge domain and procedural knowledge refers to knowledge of specific actions that are used to solve problems. This work stems from a rich foundation in mathematics education regarding the roles of procedural and conceptual knowledge in school-age children's mathematical development (Hiebert, 1986).

The issue I wish to raise in this commentary is not with the conclusions of Gabriel et al. (2013), but rather a general issue concerning the notion of conceptual and procedural *tasks* in mathematics. One of the difficulties is that a single task can reflect both types of knowledge. Indeed, consider the task of shading a geometric object (such as a square) to reflect a given fraction, say 3/4. This task appears from the outset to reflect conceptual knowledge, specifically since successful completion of the task gives some indication of the participant's knowledge of part-whole relationships in fractions. But, consider the alternative where a child is explicitly taught to perform this shading by first breaking the square into four equal sections, then shading three of the sections. Does this constitute a demonstration of conceptual knowledge? Or, does it reflect the use procedural knowledge? Hallett et al. (2010) would side with procedural knowledge since the procedure was explicitly taught to the child beforehand. Other authors (Hecht and Vagi, 2012; Gabriel et al., 2013) used this task and chose to call it a conceptual task. I would argue that the main question should not be whether the *task* is procedural or conceptual, but instead whether the employed *strategy* reflects the use of procedural or conceptual knowledge.

A related issue arises in Figure 3d of Gabriel et al. (2013). In this figure, a child has represented the fraction 2/6 by shading two parts of a circle that has been divided into six sections. The problem is that the two shaded sections are much larger than the four non-shaded sections, and as a result, the shaded fraction is actually 1/2. While this is a common error (and is apparently cross-cultural), it raises an important question. Does this child reflect conceptual understanding of fractions? I would argue no, since a conceptual understanding of the part-whole relationship would include the knowledge that the six parts should be *equal* in size. Of course, others may interpret this differently, and that is fine. My larger point is that it *can* be argued either way, and as such, the task does not define the knowledge that is used. Rather, it is the strategy used to complete the task that helps us make our conclusions.

Another common example of a task that can reflect both procedural and conceptual knowledge is the magnitude comparison task (e.g., which fraction is larger: 1/3 or 3/5?). Both Gabriel et al. (2013) and Hecht and Vagi (2012) termed this a conceptual task, presumably because it reflects the concept of fraction as a number. Indeed, if participants are forming mental representations of the fractions' magnitudes, then I feel that this is likely an accurate description. However, in a study with adults, Faulkenberry and Pierce (2011) found that on approximately 25% of trials, participants used a strategy known as cross-multiplication, where the size judgement is made by comparing cross-products (numerator of one fraction multiplied by denominator of another). This is a common procedural strategy that is employed in US schools for teaching students how to compare fractions (Boston et al., 2003). More importantly, this procedure allows the participant to arrive at an answer with no sense of the fraction as a number (i.e., no conceptual knowledge). Without explicitly asking participants to describe their solution strategy, it would not have been clear that they were using such a strategy, and by implication, it would have been impossible to know that they were using a *procedural* strategy on a task that looked conceptual.

From here, it is clear that there is a fundamental inconsistency in the literature. I think the proactive solution to this inconsistency lies in clearly delineating between the notions of conceptual/procedural *tasks* and conceptual/procedural *strategies.* Simply coining tasks as procedural or conceptual does not appear to be sufficient. Rather, we need some knowledge of a participant's solution strategy to accurately determine which type of knowledge is responsible for the solution. Admittedly, this can be difficult to ascertain, as it requires interviewing participants about their methods of solving problems. While a trial-by-trial report of strategies may be the gold standard in this type of research, such data is time-consuming to gather. More optimistically, it may be possible to get a decent measure of strategy to simply asking participants *post-hoc* to simply describe how they solve problems of a given type. At the very least, tasks that are used in studies of conceptual/procedural knowledge should be subjected to at least two rounds of independent ratings of to how they reflect one type of knowledge or the other. This was the approach used in Hallett et al. (2010), and I feel that such data should be minimally required in future studies of this type.

In summary, I believe Gabriel et al. (2013) have conducted an important study in the field of numerical cognition of fractions, particularly from the standpoint that it (1) identifies an important shortcoming of early fraction knowledge that appears to be cross-cultural, and it (2) begins an important dialogue about the methodological issues that we should consider when investigating the nature of conceptual and procedural knowledge in mathematics. We should continue to devote our time to serious investigations of the factors that influence conceptual and procedural knowledge in mathematics. At the same time, we should acknowledge that our current notion of labeling *tasks* as procedural or conceptual is limited, and that in the future we should investigate whether *strategies* employed on these tasks better reflect the use of procedural or conceptual knowledge.
